# Supplementary material for: Epistemic trust and associations with psychopathology: Validation of the German version of the Epistemic Trust, Mistrust and Credulity-Questionnaire (ETMCQ)
Source: PLoS One. 2024 Nov 14;19(11):e0312995. doi: 10.1371/journal.pone.0312995 (PMC11563411; doi:10.1371/journal.pone.0312995)
Supplement: S1 Table — (DOCX) [file pone.0312995.s001.docx]

**S1 Table. German and English items of the Epistemic Trust, Mistrust and Credulity – Questionnaire.**

| Item number  (15-item scale) | Item’s original number (18-item scale) | German item | English item | Subscale |
| --- | --- | --- | --- | --- |
| 1 | 1 | Ich frage üblicherweise andere um Rat, wenn ich  persönliche Probleme habe. | I usually ask people for advice when I have a personal problem. | Trust |
| 2 | 2 | Ich finde es leichter, Informationen zu vertrauen  und aufzunehmen, wenn sie von jemandem  stammen, der mich gut kennt. | I find information easier to trust and absorb when it comes from someone who knows me well. | Trust |
| *3* | *3* | *Ich bevorzuge, Dinge im Internet selbst herauszufinden,*  *statt andere um Informationen zu bitten.* | *I’d prefer to find things out for myself on the internet rather than asking people for information.* | *Mistrust* |
| 4 | 4 | Ich habe oft das Gefühl, dass andere nicht verstehen,  was ich will und brauche. | I often feel that people do not understand what I want and need. | Mistrust |
| 5 | 5 | Ich werde oft für naiv gehalten, weil ich fast alles  glaube, was andere mir erzählen. | I am often considered naïve because I believe almost anything that people tell me. | Credulity |
| *6* | *6* | *Wenn ich mit verschiedenen Menschen spreche,*  *kann ich mich leicht von dem überzeugen lassen,*  *was sie sagen, auch wenn dies etwas anders ist,*  *als das, was ich vorher geglaubt habe.* | *When I speak to different people, I find myself easily persuaded by what they say even if this is different from what I believed before.* | *Credulity* |
| 7 | 7 | Ein Gespräch mit Menschen, die mich schon  lange kennen, kann mir helfen, neue Perspektiven  über mich selbst zu entwickeln. | Sometimes, having a conversation with people who have known me for a long time helps me develop new perspectives about myself. | Trust |
| 8 | 8 | Ich finde es sehr nützlich, aus dem zu lernen, was  andere mir über ihre Erfahrungen erzählen. | I find it very useful to learn from what people tell me about their experiences. | Trust |
| 9 | 9 | Wenn du dem, was andere dir erzählen, zu viel  Glauben schenkst, bist du leichter verletzbar. | If you put too much faith in what people tell you, you are likely to get hurt. | Mistrust |
| 10 | 10 | Wenn mir jemand etwas erzählt, frage ich mich  sofort, warum er mir das jetzt erzählt. | When someone tells me something, my immediate reaction is to wonder why they are telling me this. | Mistrust |
| 11 | 11 | Ich habe zu oft Ratschläge von den falschen Menschen  angenommen. | I have too often taken advice from the wrong people. | Credulity |
| 12 | 12 | Verschiedene Leute haben mir gesagt, dass ich zu  leicht von anderen beeinflussbar bin. | People have told me that I am too easily influenced by others | Credulity |
| 13 | 13 | Wenn ich nicht weiß, was ich tun soll, ist mein  erster Impuls, jemanden zu fragen, dessen Meinung  ich schätze. | If I don’t know what to do, my first instinct is to ask someone whose opinion I value. | Trust |
| *14* | *16* | *Ich befolge normalerweise keinen Ratschlag, den*  *ich von anderen bekomme, selbst wenn ich*  *denke, dass der Rat wahrscheinlich gut ist.* | *I don’t usually act on advice that I get from others even when I think it’s probably sound.* | *Mistrust* |
| 15 | 17 | In der Vergangenheit habe ich falsch eingeschätzt,  wem ich glauben kann, und bin deswegen  ausgenutzt worden. | In the past, I have misjudged who to believe and been taken advantage of. | Credulity |

*Note*. Items in italic (3, 6 and 14) were removed in the German 12-Item Version of the Epistemic Trust, Mistrust and Credulity – Questionnaire. German translation by Nolte, T., Schwarzer, N., Riedl, D., Lashani, E., Kampling, H., Lampe, A., Kruse, J., Campbell, C., Montague, P.R., Fonagy, P., & Gingelmaier, S. (2023, under review). Validation of the German version of the Epistemic Trust, Mistrust and Credulity Questionnaire.
