# Supplementary material for: Epistemic trust and associations with psychopathology: Validation of the German version of the Epistemic Trust, Mistrust and Credulity-Questionnaire (ETMCQ)
Source: PLoS One. 2024 Nov 14;19(11):e0312995. doi: 10.1371/journal.pone.0312995 (PMC11563411; doi:10.1371/journal.pone.0312995)
Supplement: S2 Table — (DOCX) [file pone.0312995.s002.docx]

|  | Trust | Mistrust | Credulity |
| --- | --- | --- | --- |
| ETMCQ_1 | .747 |  |  |
| ETMCQ_2 | .603 |  |  |
| ETMCQ_7 | .554 |  |  |
| ETMCQ_8 | .532 |  |  |
| ETMCQ_13 | .694 |  |  |
| ETMCQ_3 |  | .112 |  |
| ETMCQ_14 |  | .469 |  |
| ETMCQ_4 |  | .643 |  |
| ETMCQ_9 |  | .577 |  |
| ETMCQ_10 |  | .565 |  |
| ETMCQ_5 |  |  | .521 |
| ETMCQ_6 |  |  | .304 |
| ETMCQ_11 |  |  | .818 |
| ETMCQ_12 |  |  | .655 |
| ETMCQ_15 |  |  | .699 |

**S2 Table. Factor loadings CFA model 2 (sample 1, *N* = 584).**

*Note*. All factor loadings are standardized. CFA = confirmatory factor analysis. ETMCQ = Epistemic Trust, Mistrust and Credulity - Questionnaire.
