# Supplementary material for: Epistemic trust and associations with psychopathology: Validation of the German version of the Epistemic Trust, Mistrust and Credulity-Questionnaire (ETMCQ)
Source: PLoS One. 2024 Nov 14;19(11):e0312995. doi: 10.1371/journal.pone.0312995 (PMC11563411; doi:10.1371/journal.pone.0312995)
Supplement: S3 Table — (DOCX) [file pone.0312995.s003.docx]

**S3 Table. Factor loadings for ETMCQ- and CAMSQ-items in the structural equation model (sample 1, *N* = 584).**

|  | ETMCQ factors | | | CAMSQ factors | |
| --- | --- | --- | --- | --- | --- |
|  | Trust | Mistrust | Credulity | Self-certainty | Other-certainty |
| ETMCQ_1 | .734 |  |  |  |  |
| ETMCQ_2 | .615 |  |  |  |  |
| ETMCQ_7 | .561 |  |  |  |  |
| ETMCQ_8 | .535 |  |  |  |  |
| ETMCQ_13 | .694 |  |  |  |  |
| ETMCQ_4 |  | .662 |  |  |  |
| ETMCQ_9 |  | .569 |  |  |  |
| ETMCQ_10 |  | .549 |  |  |  |
| ETMCQ_5 |  |  | .541 |  |  |
| ETMCQ_11 |  |  | .800 |  |  |
| ETMCQ_12 |  |  | .660 |  |  |
| ETMCQ_15 |  |  | .706 |  |  |
| CAMSQ_1 |  |  |  | .676 |  |
| CAMSQ_4 |  |  |  | .732 |  |
| CAMSQ_5 |  |  |  | .730 |  |
| CAMSQ_7 |  |  |  | .748 |  |
| CAMSQ_9 |  |  |  | .777 |  |
| CAMSQ_11 |  |  |  | .705 |  |
| CAMSQ_13 |  |  |  | .723 |  |
| CAMSQ_14 |  |  |  | .626 |  |
| CAMSQ_16 |  |  |  | .770 |  |
| CAMSQ_20 |  |  |  | .645 |  |
| CAMSQ_2 |  |  |  |  | .672 |
| CAMSQ_3 |  |  |  |  | .708 |
| CAMSQ_6 |  |  |  |  | .659 |
| CAMSQ_8 |  |  |  |  | .769 |
| CAMSQ_10 |  |  |  |  | .780 |
| CAMSQ_12 |  |  |  |  | .736 |
| CAMSQ_15 |  |  |  |  | .674 |
| CAMSQ_17 |  |  |  |  | .712 |
| CAMSQ_18 |  |  |  |  | .669 |
| CAMSQ_19 |  |  |  |  | .735 |

*Note.* All factor loadings are standardized. ETMCQ = Epistemic Trust, Mistrust and Credulity – Questionnaire. ETMCQ item numbering is related to the 15-item-version (Campbell et al., 2021), items 3, 6 and 14 were removed in the German 12-item-version. CAMSQ = Certainty About Mental States – Questionnaire.
