# Supplementary material for: Epistemic trust and associations with psychopathology: Validation of the German version of the Epistemic Trust, Mistrust and Credulity-Questionnaire (ETMCQ)
Source: PLoS One. 2024 Nov 14;19(11):e0312995. doi: 10.1371/journal.pone.0312995 (PMC11563411; doi:10.1371/journal.pone.0312995)
Supplement: S4 Table — (DOCX) [file pone.0312995.s004.docx]

**S5 Table A. Standardized residual correlations CFA model 1 of the Epistemic Trust, Mistrust and Credulity Questionnaire (ETMCQ).**

|  | Item_01 | Item_02 | Item_07 | Item_08 | Item_13 | *Item_03* | *Item_14* | Item_04 | Item_09 | Item_10 | Item_15 | Item_05 | *Item_06* | Item_11 | Item_12 |
| --- | --- | --- | --- | --- | --- | --- | --- | --- | --- | --- | --- | --- | --- | --- | --- |
| Item_01 | 0.000 |  |  |  |  |  |  |  |  |  |  |  |  |  |  |
| Item_02 | 0.117 | 0.000 |  |  |  |  |  |  |  |  |  |  |  |  |  |
| Item_07 | -1.500 | 0.148 | 0.000 |  |  |  |  |  |  |  |  |  |  |  |  |
| Item_08 | -1.721 | -0.592 | **3.125** | 0.000 |  |  |  |  |  |  |  |  |  |  |  |
| Item_13 | 2.248 | 0.807 | -2.186 | -1.929 | 0.000 |  |  |  |  |  |  |  |  |  |  |
| *Item_03* | -2.718 | 0.320 | 0.279 | 0.887 | -1.411 | 0.000 |  |  |  |  |  |  |  |  |  |
| *Item_14* | -2.196 | -1.125 | **-3.382** | **-4.845** | -1.386 | **4.230** | 0.000 |  |  |  |  |  |  |  |  |
| Item_04 | 1.081 | 1.226 | 0.675 | -0.980 | 0.968 | -0.677 | -0.655 | 0.000 |  |  |  |  |  |  |  |
| Item_09 | 0.076 | 0.841 | 1.776 | -0.272 | 0.568 | -0.587 | -1.734 | -0.704 | 0.000 |  |  |  |  |  |  |
| Item_10 | -0.249 | 0.733 | 1.693 | 0.669 | 1.520 | 0.663 | 0.991 | -1.662 | **3.395** | 0.000 |  |  |  |  |  |
| Item_15 | -2.387 | -0.517 | -0.807 | -1.894 | -0.791 | 0.805 | 0.412 | **5.335** | **3.459** | 0.215 | 0.000 |  |  |  |  |
| Item_05 | 1.717 | -0.070 | -0.117 | -0.588 | 1.744 | -1.848 | -2.362 | 1.964 | -2.037 | -0.670 | -3.198 | 0.000 |  |  |  |
| *Item_06* | **4.906** | 2.300 | **3.073** | **3.577** | **6.527** | -1.382 | **-3.128** | -2.295 | **-4.010** | -2.785 | **-3.036** | **4.098** | 0.000 |  |  |
| Item_11 | -2.267 | -2.590 | -2.184 | **-3.153** | -1.650 | -1.720 | 1.713 | 2.868 | **3.535** | **3.319** | **3.904** | **-5.688** | **-7.262** | 0.000 |  |
| Item_12 | 1.732 | 0.366 | -1.568 | -0.961 | 1.949 | -1.988 | -2.015 | -0.368 | -1.457 | -2.301 | **-3.958** | 2.566 | **3.119** | 0.388 | 0.000 |

*Note.* Green = trust subscale; red = mistrust subscale; yellow = credulity subscale; bold = standardized residual correlation > |3|. Items in italic (3, 6 and 14) were removed in the German 12-Item Version of the ETMCQ. CFA = confirmatory factor analysis.

**S5 Table B. Standardized residual correlations CFA model 2 of the Epistemic Trust, Mistrust and Credulity Questionnaire (ETMCQ).**

|  | Item_01 | Item_02 | Item_07 | Item_08 | Item_13 | *Item_03* | *Item_14* | Item_04 | Item_09 | Item_10 | Item_15 | Item_05 | *Item_06* | Item_11 | Item_12 |
| --- | --- | --- | --- | --- | --- | --- | --- | --- | --- | --- | --- | --- | --- | --- | --- |
| Item_01 | 0.137 |  |  |  |  |  |  |  |  |  |  |  |  |  |  |
| Item_02 | -0.978 | 0.000 |  |  |  |  |  |  |  |  |  |  |  |  |  |
| Item_07 | -0.189 | 1.766 | 0.000 |  |  |  |  |  |  |  |  |  |  |  |  |
| Item_08 | -0.387 | 0.983 | 0.000 | 0.000 |  |  |  |  |  |  |  |  |  |  |  |
| Item_13 | 1.004 | -0.032 | -1.102 | -0.791 | 0.000 |  |  |  |  |  |  |  |  |  |  |
| *Item_03* | -0.667 | 0.277 | 0.178 | 0.759 | -1.451 | 0.000 |  |  |  |  |  |  |  |  |  |
| *Item_14* | -2.371 | -1.279 | **-3.770** | **-5.073** | -1.546 | **4.421** | 0.000 |  |  |  |  |  |  |  |  |
| Item_04 | 1.321 | 1.317 | 0.142 | -1.592 | 0.976 | -0.530 | -0.176 | 0.000 |  |  |  |  |  |  |  |
| Item_09 | 0.129 | 0.836 | 1.290 | -0.644 | 0.657 | -0.555 | -2.153 | -1.412 | 0.000 |  |  |  |  |  |  |
| Item_10 | -0.340 | 0.666 | 1.222 | 0.230 | 1.694 | 0.798 | 1.184 | -1.850 | **3.269** | 0.000 |  |  |  |  |  |
| Item_15 | -1.261 | 0.554 | 0.419 | -0.759 | 0.395 | 0.487 | -1.215 | **3.574** | 1.159 | -2.331 | 0.000 |  |  |  |  |
| Item_05 | **3.199** | 1.359 | 1.394 | 0.981 | **3.150** | -1.606 | -1.310 | **3.399** | -1.272 | 0.491 | 0.225 | 0.000 |  |  |  |
| *Item_06* | **5.749** | **3.068** | **3.797** | **4.304** | **7.197** | -1.067 | -1.671 | 0.374 | -2.530 | -0.938 | 1.403 | 0.000 | 0.000 |  |  |
| Item_11 | -1.002 | -1.538 | -0.861 | -2.099 | -0.495 | -2.276 | -0.654 | -0.141 | -0.100 | 0.337 | 0.425 | -2.226 | -1.383 | 0.000 |  |
| Item_12 | **3.711** | 2.016 | 0.456 | 0.934 | **3.756** | -1.878 | -1.370 | 0.631 | -1.267 | -1.709 | -1.973 | 0.000 | 0.000 | 1.699 | 0.000 |

*Note*. Green = trust subscale; red = mistrust subscale; yellow = credulity subscale; bold = standardized residual correlation > |3|. Items in italic (3, 6 and 14) were removed in the German 12-Item Version of the ETMCQ. CFA = confirmatory factor analysis. Correlated residuals as in Campbell et al. (2021): Item 7 ~~ item 8; item 3 ~~ Item 1; item 5 ~~ item 6; item 5 ~~ item 12; item 6 ~~ item 12.

**S5 Table C. Standardized residual correlations CFA model 4 of the Epistemic Trust, Mistrust and Credulity Questionnaire (ETMCQ).**

|  | Item_01 | Item_02 | Item_07 | Item_08 | Item_13 | Item_04 | Item_09 | Item_10 | Item_15 | Item_05 | Item_11 | Item_12 |
| --- | --- | --- | --- | --- | --- | --- | --- | --- | --- | --- | --- | --- |
| Item_01 | 0.000 |  |  |  |  |  |  |  |  |  |  |  |
| Item_02 | -1.615 | 0.000 |  |  |  |  |  |  |  |  |  |  |
| Item_07 | -0.190 | 2.172 | 0.000 |  |  |  |  |  |  |  |  |  |
| Item_08 | -0.365 | 1.296 | 0.000 | 0.000 |  |  |  |  |  |  |  |  |
| Item_13 | 1.101 | -0.191 | -1.756 | -1.101 | 0.000 |  |  |  |  |  |  |  |
| Item_04 | 0.363 | 0.577 | -0.330 | -2.079 | 0.354 | 0.000 |  |  |  |  |  |  |
| Item_09 | -0.575 | 0.346 | 0.886 | -1.038 | 0.097 | -2.279 | 0.000 |  |  |  |  |  |
| Item_10 | -1.109 | 0.144 | 0.717 | -0.224 | 1.032 | -1.690 | **3.394** | 0.000 |  |  |  |  |
| Item_15 | -1.538 | 0.368 | 0.264 | -0.914 | 0.142 | **3.464** | 0.620 | -2.587 | 0.000 |  |  |  |
| Item_05 | **3.013** | 1.214 | 1.286 | 0.880 | 2.987 | **3.251** | -1.722 | 0.459 | -0.087 | 0.000 |  |  |
| Item_11 | -1.403 | -1.748 | -1.049 | -2.294 | -0.837 | -0.616 | -0.765 | 0.551 | 0.478 | -2.301 | 0.000 |  |
| Item_12 | **3.491** | 1.835 | 0.320 | 0.810 | **3.606** | 0.386 | -1.774 | -1.763 | -2.349 | 0.000 | 2.021 | 0.000 |

*Note*. Green = trust subscale; red = mistrust subscale; yellow = credulity subscale; bold = standardized residual correlation > |3|. CFA = confirmatory factor analysis. Allowed correlated residuals in the model specifications: Item 7 ~~ Item 8; Item 5 ~~ Item 12.
