# Supplementary material for: Epistemic trust and associations with psychopathology: Validation of the German version of the Epistemic Trust, Mistrust and Credulity-Questionnaire (ETMCQ)
Source: PLoS One. 2024 Nov 14;19(11):e0312995. doi: 10.1371/journal.pone.0312995 (PMC11563411; doi:10.1371/journal.pone.0312995)
Supplement: Attachment — Submitted filename: Review PONE-D-23-22407R1.pdf [file pone.0312995.s008.pdf]

Review PONE-D-23-22407R1

SEM analysis: p13. The authors speak of a 5-factor model they have fit, and then they seem to talk about sth else and report a very high value of multiv. skewness for a CFA of the CAMSQ scale. The value of the multiv. skewness appears way too high to simply continue with an analysis of a SEM model. MLR estimation probably cannot cope with having such a large value of skewness in the data. This makes the results very doubtful in its entirety. The authors should clearly report on the skewness of the separate indicator variables. Then, depending on whether that there continue to be some extremely skewed indicators, they cannot simply proceed with the SEM analysis. MLR estimation still requires skewness to lie within some moderate range. So, it has to be clarified if one could at all proceed with SEM from here on. A problem is also the poor statistical model fit of model 1. What explains it? Is the theory wrong, or does the sample have characteristics that would lead to it? What does the residual correlation matrix say? For a revision of the paper, these questions should be clearly addressed.

Model 2: How large were the correlations among the residuals? The interpretation of the results depend on this.

Model 4: Factor 2 and 3 were highly correlated, which speaks perhaps for 2-factor model rather than 3 factors. Could that be a consequence of the shortening of the questionnaire? The shortening appears to have been made based on the original data (it's just data-fitting by changing the instrument) without cross-validation. An actual argument for a replication of Campbell's factor structure would have to go into a cross-validation here, by pulling another sample and fitting it both to the 15-item version and the shortened 12-item version.

Discriminant validity: The sample size for sample 2 is just too small right from the start to address the research question. I believe it should be taken out from the manuscript.
