# Supplementary material for: Epistemic trust and associations with psychopathology: Validation of the German version of the Epistemic Trust, Mistrust and Credulity-Questionnaire (ETMCQ)
Source: PLoS One. 2024 Nov 14;19(11):e0312995. doi: 10.1371/journal.pone.0312995 (PMC11563411; doi:10.1371/journal.pone.0312995)
Supplement: Attachment — Submitted filename: Review PONE-D-23-22407_final.docx [file pone.0312995.s006.docx]

**Review PONE-D-23-22407**

The paper titled "Epistemic Trust and Associations with Psychopathology: Validation of the German Version of the Epistemic Trust, Mistrust, and Credulity-Questionnaire (ETMCQ)" aims to validate the self-report measure ETMCQ in the German population and explores the concept of epistemic trust and its potential associations with psychopathology. While the study certainly has some strengths in addressing an important area of research and contributes to the field of studies on epistemic trust and its dimensions, it has some notable weaknesses that warrant careful consideration.

The paper may contribute to cross-cultural comparison, as the ETMCQ was validated in different populations. Moreover, the author’s effort to use a transdiagnostic approach when discussing and studying ET and psychopathology is appreciable. However, the study presents some issues and lack of transparency regarding the factorial validity of the scale. These weaknesses currently hinder the publication's consideration. Further refinement and psychometric investigation of the data is vital to improve the paper.

Here the editor and the authors may find specific comments with respect to some aspects of the paper that need to be tackled. Addressing these issues would enhance the overall presentation and impact of the research findings.

Therefore, my main reservations concern the research design and, consequently, the method and result sections. In more details:

- In the manuscript, the authors refer to “the first investigation of the validity of the German version [29], partly deviating results of the factor structure compared to Campbell et al. [11]” (see page 6, lines 130-134). The reference of Nolte [29] is reported as follows: Nolte T. [German version of the Epistemic Trust, Mistrust and Credulity – Questionnaire]. Forthcoming. It appears to be another study validating the same instrument in the same language; hence, I believe it would be helpful if the authors can specify if and how their work differs from the previous one. More importantly, since they follow its proposed 12-item version for the ETMCQ, the reader should have access to the data of first paper for a comprehensive understanding. If the data is not publicly accessible yet, the authors should consider waiting until this paper is published. Furthermore, another point on which further clarification is needed is why the authors chose to use the 18-item version of the ETMCQ for their analyses when in the original validation of the instrument the items 16, 17, and 18 were excluded.
- Page 12, lines 280-283. In the paragraph of “Statistical Analysis”, the authors state that “In the third and fourth model, three items with the lowest factor loadings were removed and a 12-item version of the ETMCQ without and with correlated residuals was tested [29]”. In addition to the problems created by the unavailability of a readable reference to Nolte’s paper, which the authors repeatedly cite, it would be advisable to include the results of the PCA (or EFA), so that the factor loadings of each of the items are available to the reader. This is particularly crucial given that the Confirmatory Factor Analysis (CFA) reported by the authors appear to include items (i.e., item 16, 17, and 18) that were previously excluded from the ETMCQ, both in the original validation by Campbell and colleagues and in other currently published validations (e.g., the Italian one by Liotti and colleagues). Including PCA or EFA results would provide valuable insights into the factor structure and the retained items’ suitability for the German version of the ETMCQ.
- The use of standardized residuals in the CFA for a fairly new instrument like the ETMCQ should be approached with caution; it would be advisable for the authors to provide more support for their decision, especially since they end up validating a different version of the instrument than the original one (Campbell et al. 2021), as they used some item excluded in the original version.
- The results regarding construct validity should be described more thoroughly, and the authors should make assumptions about their significance based on the existing literature (for example, the relationship between epistemic mistrust and other certainty as measured by the CAMSQ, or the positive relationship between epistemic trust and negative affectivity could be discussed more exhaustively).
- The inter-scale correlation between epistemic mistrust (EM) and credulity (EC) is notably high. This result seems to indicate that there is a significant degree of overlap, or shared variance, between the two scales. Again, while it is possible to hypothesize that EM and EC may coexist in the same individual, the two ETMCQ subscales are theoretically expected to represent fundamentally opposite attitudes toward interpersonally transmitted information. It is not clear how the authors justify such a strong correlation. Moreover, it’s reasonable to assume that the coexistence of mistrust and credulity (i.e., two maladaptive stances) would be more likely to be found in psychopathological subjects. However, the sample in this study is purportedly normative. This finding warrants further discussion and examination throughout the article to better elucidate its implications and potential theoretical or methodological considerations.
- Moreover, in the method section the authors specify that for correlations they used Spearman and that they used Wilcoxon rank test for group differences. I think they should briefly clarify why they chose to use these non-parametric methods.
- The authors conducted a t-test to examine the difference between clinical and non-clinical samples with respect to the ETMCQ domains on 30 participants per group, which is considered the minimum sample size to conduct this analysis. Even if the differences between groups are not significative, I think the authors should have computed a power analysis (for example, using GPower), to estimate the effect size and specify what was the probability to find the effect, considering that the authors hypothesized its existence. The power analysis results should be reported. Moreover, given that with an independent samples t-test conducted on a few participants the minimal effect expected to be found is a large effect, the authors should specify (even if only in a footnote) that this might be the reason why this result is not significant. The need for research on the difference between clinical and non-clinical samples regarding epistemic trust may be explored in the Implications and future directions section.

Other critical issues that should be addressed are reported below.

Specifically, in the “Introduction” section:

- Pag. 3, lines 45-46. “Epistemic trust (ET) refers to an individual's willingness to perceive signals sent by others as relevant, trustworthy, and generalizable to other contexts”. Here I would say “transmitted” instead of “sent” and I would substitute “signals” with “information”, as this terminology seems more appropriate to describe epistemic trust rightfully.
- Page 3, lines 60-61. “Therefore, epistemic vigilance acts as a protective mechanism to question possible misinformation”. Here I would explain in more detail what epistemic vigilance is, since it is a pivotal concept for the theory of ET.
- Page 4*,* lines 69-74. Here there is some ambiguity regarding whether the authors are asserting that “feelings of isolation,” often linked with epistemic mistrust, subsequently lead to epistemic hunger, ultimately resulting in epistemic credulity. While it is plausible that individuals with elevated psychopathological tendencies and a history of adverse experiences (e.g., individuals with BPD) may exhibit a coexistence of both mistrust and credulity, it would be more precise to acknowledge that, regardless of whether they co-occur within the same individual, both mistrust and credulity are recognized in the literature on epistemic trust as maladaptive epistemic stances stemming from traumatic experiences, particularly during childhood (as subsequently elaborated upon in the article). The current phrasing of the sentence might inadvertently convey the idea that mistrust serves as the precursor to credulity through the mechanism of epistemic hunger.
- Pag. 7*,* lines 149–168. To the point about hypotheses and aims, it would be helpful if the authors provided further clarification on their expectations concerning the relationship between sociodemographic characteristics and the three subscales of the ETMCQ.

In the “Discussion” section of the manuscript:

- Page 22, lines 462-467. “The high correlative relationship between these two dimensions (*mistrust and credulity*) can be substantiated by the assumption that in the absence of ET, rapid shifts between credulity and hypervigilance could exist, therefore the two factors are closely related to each other. However, this interpretation raises the question of whether the mistrust subscale measures hypervigilance rather than persistent epistemic mistrust as a result of developmental adaptation to an adverse social environment”. The concept and term “epistemic mistrust” coincides with a rigid and all-encompassing epistemic hypervigilance, hence, the reason why the authors contrast mistrust with epistemic hypervigilance rather than epistemic vigilance is puzzling to me. Perhaps this section could benefit from further elaboration to clarify the rationale behind this comparison.

Overall, I would like to highlight the need for a proofreading or a thorough revision of the English language, as to improve clarity and readability.
