# Supplementary material for: Epistemic trust and associations with psychopathology: Validation of the German version of the Epistemic Trust, Mistrust and Credulity-Questionnaire (ETMCQ)
Source: PLoS One. 2024 Nov 14;19(11):e0312995. doi: 10.1371/journal.pone.0312995 (PMC11563411; doi:10.1371/journal.pone.0312995)
Supplement: Attachment — Submitted filename: Response to Reviewers.docx [file pone.0312995.s007.docx]

Hanna Landenmark
Staff Editor
PLOS ONE

Dear Ms. Landenmark,

Herewith I am submitting a revised version of our manuscript **“Epistemic trust and associations with psychopathology: Validation of the German version of the Epistemic Trust, Mistrust and Credulity-Questionnaire (ETMCQ)”** to *PLOS ONE* on behalf of all authors.

We would like to thank you for giving us the opportunity to submit a revised version of our manuscript and also for your and the reviewer’s very helpful comments to improve the manuscript.

Please find below a point-by-point response to the reviewer’s comments, which we have incorporated in the manuscript (changes are highlighted in the main text).

The manuscript was prepared in accordance with PLOS ONE Style guidelines. The manuscript has not previously been published or is being considered for publication elsewhere. There was no conflict of interest for any of the authors of the article.

We hope that all revision points have been addressed satisfactorily and the manuscript now meets the high standards for publication in PLOS ONE and look forward to hearing from you.

Sincerely,

Anna-Maria Weiland

**Reply to the reviewer comments:**

**Editor (E):** Please see the comments from two reviewers below. Please note especially that reviewer 2 queries whether the German translated questionnaire is available, and whether any studies noted as "forthcoming" have already performed a validation of this questionnaire. Please clearly outline in your response whether the translated questionnaire and the forthcoming studies are available, and how other researchers may access these.

We note that some of the reviewer comments may be seen a little terse in tone in places, for which we apologize, but overall, I hope that the comments are useful to you in order to revise the manuscript and strengthen the study.

**Author reply (AR):** Thank you very much for your appreciation of our study and the opportunity to submit a revised version of our manuscript. Below, we provide comments on each point raised by the reviewers. Additionally, as recommended by you, we have attached the German translation of the questionnaire as a document in the appendix.

**Reviewer #1: ID: PONE-D-23-22407**

Introduction
**(R#1):** Line 56-59, Page 3: What are the further conceptual groundwork proposed by Sperber et al. and Wilson and Sperber? This is what needs to be clarified.

**(AR):** Thank you for this suggestion. To enhance clarity in the formulation, we have added relevant information in lines 55-60, page 3 and rephrased the text. Sperber et al. and Wilson and Sperber have explored thoroughly the concept of epistemic vigilance in human communication, and Fonagy et al. consider epistemic vigilance as an adaptive counterbalance to (blind) trust.
**(R#1):** Paragraph 1, Page 3-4: So, on what basis, does the authors focu on such a psychosocial phenomenon? Are there any relevant statistics to support such a psychological problem on a social level?

**(AR):** Thank you for your comment. Some aspects discussed in the framework of epistemic trust have only been theoretically described, and empirical data on this topic is still limited. In this paragraph, we cited the few available empirical studies [11, 15, 16] that describe initial findings supporting the line of thinking provided by Fonagy and colleagues. Therefore, the development of the ETMCQ is an important step towards filling this research gap. Three recently published articles were integrated into the discussion (lines 630-640) to illustrate the importance of the ET framework as a clinically and socially relevant phenomenon.
**(R#1):** Line 80-86, Page 4: How strong is the association? In addition, the authors give many new concepts in this paragraph, and if the authors do not intend to elaborate in depth, I would suggest streamlining unnecessary details to minimize distraction for the reader.

**(AR):** Thank you for pointing this out. We rephrased and streamlined this paragraph (line 75-94, page 4-5) and now we provide information about the direction and strength of the associations between epistemic trust, mistrust and credulity and the related constructs, especially mentalizing (line 85-87) and adverse childhood experiences (90-94).
**(R#1):** Line 94-99, Page 4-5: What is the so-called promising initial empirical evidence? I wish the authors could be more specific in the content and not be vague.

**(AR):** Thank you for raising this point. To be more specific we paraphrased this sentence and briefly describe the main results of the cited studies regarding the association between mistrust, credulity and mentalizing (Line 85-87): “In this regard, Campbell et al. [11] and Liotti et al. [15] provide initial empirical evidence indicating positive associations between mistrust, credulity, and difficulties in understanding mental states.”
**(R#1):** Line 100-107, Page 5: Does the current content argue that ET is predictive of mental disorders? Or is there any other authoritative literature that apparently gives this conclusion?

**(AR):** The introduction describes that there is currently still an empirical research gap on the link between ET and psychopathology (line 95-110). We have now added a sentence to emphasize that these assumptions are primarily theoretical (line 97-98). In the following three sentences, we have cited three empirical studies [17, 11, 16] that describe initial findings supporting the line of thinking provided by Fonagy and colleagues.
**(R#1):** Line 121-135, Page 6: Have the authors looked at the measurement properties of other language versions of the ETMCQ? This is important for the cross-cultural validation status of this scale.

**(AR):** Thank you for this important question. In line 121-126, we address the two currently available validation studies of the ETMCQ. We summarize the results of the original English validation by Campbell et al. (2021) in line 121-123, and briefly discuss the Italian validation study by Liotti et al. (2023) [15] in line 123-126.
**(R#1):** Overall: The authors need further discretion in the background of the research, and providing relevant data would be a more intuitive approach. Also, the authors need to be more precise and cohesive in their presentation.

**(AR):** We have thoroughly revised the introduction, making it more precise and cohesive and also shortening some parts to streamline it. So far, some aspects discussed in the framework of epistemic trust, in particular the link between ET and psychopathology, have only been theoretically described, and empirical data on this topic is still limited. Therefore, the development of the ETMCQ is an important step towards filling this research gap.

Methods
Participants and Procedures
**(R#1):** 1) The sample information needs to be further refined, e.g. from which region did the sample come? What were the inclusion and exclusion criteria for the sample? Is the sample representative of the general population?

**(AR):** Thank you for bringing this point to our attention and give us the possibility to clarify. As described in the preregistration (<https://doi.org/10.23668/psycharchives.5406>; cited in line 159-160, p.7), only participants who met the following inclusion criteria were included: Age between 18 - and 74 years, capable of giving informed consent, sufficient understanding of the German language (included countries for recruitment were Germany, Austria, & Switzerland). No other exclusion or inclusion criteria existed. To be more precise in the description, we changed “German population” in “German-speaking population” in line 26 in the abstract. The sample is only roughly representative of the general population of Germany, because of a slightly higher mean age (M = 50.9) and a higher proportion of females (63%) compared to population distributions of the most recent German census conducted in 2011 (assuming: female (51.2%); (https://ergebnisse2011.zensus2022.de).

**(R#1):** 2) Authors should also describe sample collection procedures, e.g. how was the sample size determined? How were the questionnaires distributed and collected? How was quality control performed, etc., whether in detail or briefly, I think this is necessary to describe a study’s methodology.

**(AR):** Our study is part of a larger research project, and a revised version of the associated article (cited in line 165) has been submitted. Therefore, we added the following description of our study procedures in the revised version of the manuscript (line 167-170: “Sample 1 (*N* = 584) was recruited online using the German panel provider Respondi and funded by PsychLab (service of the Leibniz Institute for Psychology). The initial target sample size (*n* = 490) was based on recommendations to obtain error-free correlation estimates by Kretzschmar and Gignac [29].”)

Regarding the question on quality control: as described in the preregistration (<https://doi.org/10.23668/psycharchives.5406>) and in line 170-172, the questionnaires in this study were presented in a forced-choice format and therefore no missing data has been incurred. To screen for careless responding, the relative speed index was calculated, a reliable indicator for detecting meaningless data based on participants’ completion times. Participants who responded at least twice as fast as the average has subsequently been removed. Furthermore, we implemented an attention check item to ensure data quality. No participants have been removed based on univariate or multivariate normality. In the “Statistical Analysis” section, we have now included a reference to the preregistration to make the data cleansing strategies available (line 250-252).

**(R#1):** 3) The sample size for Sample 2 is so small that the representativeness of the results is questionable.

**(AR):** Thank you for mentioning this point. In our discussion (line 469-470), we expressed the following regarding this issue: “However, because these differences were not significant, and the sample size was relatively small, a random result cannot be ruled out.”

Statistical Analysis
**(R#1):** Line 301-314, Page 13: Effect size on outcome should be moved to the results section.

**(AR):** Unfortunately, we are not sure which effect sizes you are referring to. You were referring to line 301-314 (revised manuscript line 290-306) on page 13, where we described the applied statistical procedures for our Structural Equation Model (SEM) and reported the results of prerequisite analysis, that are needed to conduct a SEM and to comprehensibly illustrate why estimation methods like MLR were used. Therefore, we would prefer to keep this in the method section.

Results
**(R#1):** 1) I would suggest the authors to adjust the tables to a three line table format, also I think the font in the NOTE should be separate from the main text.

**(AR):** Thank you for your comment. We have aligned the style and format of the tables and table legends with the style and format guidelines of PLOS ONE. No variations in font styles are recommended in these guidelines. Also, the PLOS ONE manuscript formatting guidelines state a cell-based table format with all vertical and horizontal lines.
**(R#1):** 2) Only important results need to be described in text, otherwise what is the purpose of the table? This problem of redundancy is particularly evident in the section on Associations with demographic features.

**(AR):** We have thoroughly checked the results section for redundancies. In the section “ETMCQ factor structure” (line 358-365), we removed the redundant mention of the fit indices’ values for model 4. We left the results for the correlations between the latent factors in the text because they also give the exact p-values, which do not appear in the figure. For the same reason, we would prefer to keep the results on the SEM in the text (paragraph “Mentalization and epistemic trust”, line 416-434). In line 331-356 we described the results on the associations between ETMCQ-subscales and demographic features only in the text and no table additionally displays these results.
**(R#1):** 3) The results of some of the model fits in Table 2 are not very ideal, did the authors consider further factorial constraints.

**(AR):** Thank you very much for your comment. Table 2 shows the results of our confirmatory factor analysis in which we tested four models with different factorial constraints (correlated residuals, deleted items). The fourth model with 12-items and correlated residuals showed good model fit indices and therefore we did not consider further factorial constraints.
**(R#1):** 4) The figures in the appendix are missing titles and notes.

**(AR):** Thank you very much for your comment. Hopefully we can resolve the misunderstanding with our explanation: We followed the PLOS ONE figure guidelines, indicating that “each figure must be prepared and submitted as an individual file” and “figure captions must be inserted in the text of the manuscript, immediately following the paragraph in which the figure is first cited (read order). Do not include captions as part of the figure files themselves or submit them in a separate document.”. Therefore, our figure captions (including titles and notes) can be found in Line 372-375 and 436-439.

Discussion
**(R#1):** 1) On page 15, lines 337-338, the authors state that “compared to a representative sample of the German general population, sample 1 showed a higher burden”. What is the reason for such a difference?

**(AR):** Thank you for your important comment. To address your question, we added a brief discussion about this topic in our limitations (line 588-595): “Second, sample 1 reported more experiences of traumatic events compared to a representative sample of the German population [47]. One possible explanation for this could be the higher mean age in our sample, as older age is associated with more experience of physical neglect [62]. Another explanation for more traumatic childhood experiences in sample 1 might be a self-selection bias, which has been reported in other studies [63].”
**(R#1):** 2) Based on the main points and length of the authors’ discussion, I would suggest that the authors further refine their language, incorporate more of the relevant literature, and explain and extend inferences from the study’s findings. Honestly the content now makes it a struggle for me to read.

**(AR):** We have carefully revised the discussion, improved its precision and cohesiveness and also streamlined it.
Implications and future directions
**(R#1):** 1) I’m not sure how the authors considered this part, however it is apparently that this section looks more like a discussion of the results than the impact of the outcomes of this study.

**(AR):** Thank you for addressing this point. We revised the section “Implications and future directions” and streamlined the content.
**(R#1):** 2) I don’t see any future research directions planned by the authors in this section, and in conjunction with the first sentence of the first paragraph, perhaps the authors are forgetting to add this content.

**(AR):** Thank you for your comment. We have revised the section “Implications and future directions” and streamlined the content: we mention some future research directions when discussing our results: Regarding further investigation of the ETMCQ-structure (line 464-465), longitudinal studies and studies with larger clinical samples (line 474-477; 551-553; 604-605), a possible replication of observed patterns in different samples (line 543-545), future research on the relationships between the ETMCQ and the accuracy of mentalizing ability and the relationship with hypermentalizing (line 572-579). Additionally, we address the need for future multimethod approaches to examine the construct validity of the ETMCQ (line 572-576; 605-607), the need for cross-cultural studies (line 609-611), that future research should examine the distinctions and connections to other trust concepts (line 613-615). Finally, in line 623-628, we highlight the potential significant role of the ETMCQ to implement and evaluate therapeutic interventions that establish or re-establish ET.

Conclusion
**(R#1):** The conclusion of the study should provide an overview of the background, content and methodology of the study, followed by a list of the main conclusions, and finally a full-text summary or an outlook for future research. The current content is not a conclusion, but rather like a brief introduction. Authors need to read the relevant literature and learn how to write a competent conclusion.

**(AR):** Thank you for emphasizing the importance of precision and clarity, particularly in the conclusion. We have revised the conclusion to provide an improved summary of the study's objectives, main results, and future research outlook (line 634 – 644).

**Reviewer #2**

**(R#2):** The paper titled "Epistemic Trust and Associations with Psychopathology: Validation of the German Version of the Epistemic Trust, Mistrust, and Credulity-Questionnaire (ETMCQ)" aims to validate the self-report measure ETMCQ in the German population and explores the concept of epistemic trust and its potential associations with psychopathology. While the study certainly has some strengths in addressing an important area of research and contributes to the field of studies on epistemic trust and its dimensions, it has some notable weaknesses that warrant careful consideration.
The paper may contribute to cross-cultural comparison, as the ETMCQ was validated in different populations. Moreover, the author’s effort to use a transdiagnostic approach when discussing and studying ET and psychopathology is appreciable. However, the study presents some issues and lack of transparency regarding the factorial validity of the scale. These weaknesses currently hinder the publication's consideration. Further refinement and psychometric investigation of the data is vital to improve the paper.
Here the editor and the authors may find specific comments with respect to some aspects of the paper that need to be tackled. Addressing these issues would enhance the overall presentation and impact of the research findings.
Therefore, my main reservations concern the research design and, consequently, the method and result sections. In more details:
**(AR):** We would like to thank the reviewer for his/her time and the appreciation of our study. Your feedback has been very helpful, and we address each of the comments below.

**(R#2):** In the manuscript, the authors refer to “the first investigation of the validity of the German version [29], partly deviating results of the factor structure compared to Campbell et al. [11]” (see page 6, lines 130-134). The reference of Nolte [29] is reported as follows: Nolte T. [German version of the Epistemic Trust, Mistrust and Credulity – Questionnaire]. Forthcoming. It appears to be another study validating the same instrument in the same language; hence, I believe it would be helpful if the authors can specify if and how their work differs from the previous one. More importantly, since they follow its proposed 12-item version for the ETMCQ, the reader should have access to the data of first paper for a comprehensive understanding. If the data is not publicly accessible yet, the authors should consider waiting until this paper is published. Furthermore, another point on which further clarification is needed is why the authors chose to use the 18-item version of the ETMCQ for their analyses when in the original validation of the instrument the items 16, 17, and 18 were excluded.

**(AR):** Thank you very much for your valuable comments. First, we have now added the updated Nolte et al. reference [32] to the reference list. As the reviewer is correctly pointing out, Nolte et al.’s paper is another study validating the German version of the ETMCQ. Our study is not related to Nolte et al. and is also the first study that investigates associations of the ETMCQ with psychopathology and specifically personality functioning and maladaptive traits, as well as the first study to examine the ETMCQ in a clinical and a non-clinical sample. As the Nolte et al. study is currently still under review, we now describe our methodology independently from Nolte et al. and explain our selected CFA models (see line 274-278). To illustrate the removal of the three items from the original 15-item version, we have added an additional table with the factor loadings to the Supplement (see S2 Table_CFA Factor Loadings Model 2).
Furthermore, as the item numbering seems to have been misleading, we now adopt the item numbering from Campbell et al.'s 15-item version in our revised manuscript and the tables to provide more clarity. We would like to emphasize that we used the same items as Campbell and colleagues (and Liotti and colleagues as well) in the 15-item version. In order to compare the items in both languages, we have now included a table with the items translated into German in the supplement (see S1 Table_German and English ETMCQ items).

**(R#2):** Page 12, lines 280-283. In the paragraph of “Statistical Analysis”, the authors state that “In the third and fourth model, three items with the lowest factor loadings were removed and a 12-item version of the ETMCQ without and with correlated residuals was tested [29]”. In addition to the problems created by the unavailability of a readable reference to Nolte’s paper, which the authors repeatedly cite, it would be advisable to include the results of the PCA (or EFA), so that the factor loadings of each of the items are available to the reader. This is particularly crucial given that the Confirmatory Factor Analysis (CFA) reported by the authors appear to include items (i.e., item 16, 17, and 18) that were previously excluded from the ETMCQ, both in the original validation by Campbell and colleagues and in other currently published validations (e.g., the Italian one by Liotti and colleagues). Including PCA or EFA results would provide valuable insights into the factor structure and the retained items’ suitability for the German version of the ETMCQ.

**(AR):** Thank you again for this comment. As explained in our previous answer, we no longer refer to the article by Nolte and colleagues and explain our selected CFA models comprehensively (see changes in the manuscript in the paragraph of “Statistical analysis” line 274-278 and in the results section line 358-365).

**(R#2):** The use of standardized residuals in the CFA for a fairly new instrument like the ETMCQ should be approached with caution; it would be advisable for the authors to provide more support for their decision, especially since they end up validating a different version of the instrument than the original one (Campbell et al. 2021), as they used some item excluded in the original version.

**(AR):** Thank you for pointing this out. As mentioned earlier, the item numbering seems to have been misleading and we now adopt the item numbering from Campbell et al.'s 15-item version in our revised manuscript and the tables to provide more clarity. We would like to emphasize again, that we used the same items as Campbell and colleagues in the 15-item version. And maybe, there is another misunderstanding, but we did not use standardized residuals in the CFA. We reported the standardized factor loadings (see the figure caption for Fig 1 in line 372-375) and used the same correlated residuals as Campbell and colleagues in their 15-item model.

**(R#2):** The results regarding construct validity should be described more thoroughly, and the authors should make assumptions about their significance based on the existing literature (for example, the relationship between epistemic mistrust and other certainty as measured by the CAMSQ, or the positive relationship between epistemic trust and negative affectivity could be discussed more exhaustively).

**(AR):** Thank you for your comment. We have now included a possible explanation for the positive relationship between epistemic trust and negative affectivity in lines 525-528 of the revised manuscript, where we outline the controversial informative value of the negative affectivity trait domain [Bach et al., 2022]. In lines 538-548, we discussed the default mode considerations regarding ET, the heterogenous findings of relationships and the need for “further examination of these phenomena in additional samples to provide insight into whether ET can be considered a resilience factor in specific areas or if the trust subscale does not fully capture the construct.”.
The relationship between mistrust and other-certainty (CAMSQ) is discussed in lines 563-582.

**(R#2):** The inter-scale correlation between epistemic mistrust (EM) and credulity (EC) is notably high. This result seems to indicate that there is a significant degree of overlap, or shared variance, between the two scales. Again, while it is possible to hypothesize that EM and EC may coexist in the same individual, the two ETMCQ subscales are theoretically expected to represent fundamentally opposite attitudes toward interpersonally transmitted information. It is not clear how the authors justify such a strong correlation. Moreover, it’s reasonable to assume that the coexistence of mistrust and credulity (i.e., two maladaptive stances) would be more likely to be found in psychopathological subjects. However, the sample in this study is purportedly normative. This finding warrants further discussion and examination throughout the article to better elucidate its implications and potential theoretical or methodological considerations.

**(AR):** Thank you for your comment. The close relationship between mistrust and credulity is justified by the different correlational patterns found with the trust subscale (see lines 459-465) and other related constructs. For example, in lines 515-518, we concluded that maladaptive personality traits are associated with both mistrust and credulity, indicating that these are independent, learned strategies for social communication. In terms of methodology, we highlighted in lines 461-465 the need to consider the item reduction of the mistrust subscale and emphasized the importance of further investigations into the ETMCQ factor structure. The paragraph titled 'Implications and future directions' and the conclusion have been significantly revised to present the points more clearly.

**(R#2):** Moreover, in the method section the authors specify that for correlations they used Spearman and that they used Wilcoxon rank test for group differences. I think they should briefly clarify why they chose to use these non-parametric methods.

**(AR):** Thank you for pointing this out. Non-parametric methods were used due to the non-normal distribution of the ETMCQ data, as described in lines 260-263. We included a brief explanation of our decision in the referred paragraph of the method section (line 255-257: “Due to the non-normal distribution of the data, non-parametric Wilcoxon rank tests for two independent samples, Spearman correlations, and analysis of variances (ANOVAs) were conducted.”)
**(R#2):** The authors conducted a t-test to examine the difference between clinical and non-clinical samples with respect to the ETMCQ domains on 30 participants per group, which is considered the minimum sample size to conduct this analysis. Even if the differences between groups are not significative, I think the authors should have computed a power analysis (for example, using GPower), to estimate the effect size and specify what was the probability to find the effect, considering that the authors hypothesized its existence. The power analysis results should be reported. Moreover, given that with an independent samples t-test conducted on a few participants the minimal effect expected to be found is a large effect, the authors should specify (even if only in a footnote) that this might be the reason why this result is not significant. The need for research on the difference between clinical and non-clinical samples regarding epistemic trust may be explored in the Implications and future directions section.

**(AR):** Thank you for your valuable comment. The sample size planning was based on Cohen's (1988) general recommendations for t-tests, a medium effect size and α = .05 with N = 50 or 64. The final decision to recruit at least 30 participants per group was also based on research economic reasons. Because our study is the first to examine the ETMCQ in a clinical and a non-clinical sample there were no other related studies to extract an estimated effect size to compute an a priori power analysis with G*Power. In our discussion (line 469-470), we expressed the following regarding this issue: “However, because these differences were not significant, and the sample size was relatively small, a random result cannot be ruled out.”. And in line 473-477 we discussed that “future research should therefore examine the differential strength of the ETMCQ with psychopathology in a larger sample either with no or at the beginning of treatment.”.

Other critical issues that should be addressed are reported below.
Specifically, in the “Introduction” section:
**(R#2):** Pag. 3, lines 45-46. “Epistemic trust (ET) refers to an individual's willingness to perceive signals sent by others as relevant, trustworthy, and generalizable to other contexts”. Here I would say “transmitted” instead of “sent” and I would substitute “signals” with “information”, as this terminology seems more appropriate to describe epistemic trust rightfully.

**(AR):** Thank you for this helpful comment. We incorporated these suggestions (line 44-45, page 3).

**(R#2):** Page 3, lines 60-61. “Therefore, epistemic vigilance acts as a protective mechanism to question possible misinformation”. Here I would explain in more detail what epistemic vigilance is, since it is a pivotal concept for the theory of ET.
**(AR):** Thank you for addressing this important point. We rephrased this paragraph and described epistemic vigilance as the cognitive ability of questioning the reliability of a source of information (line 55-62, page 3).

**(R#2):** Page 4, lines 69-74. Here there is some ambiguity regarding whether the authors are asserting that “feelings of isolation,” often linked with epistemic mistrust, subsequently lead to epistemic hunger, ultimately resulting in epistemic credulity. While it is plausible that individuals with elevated psychopathological tendencies and a history of adverse experiences (e.g., individuals with BPD) may exhibit a coexistence of both mistrust and credulity, it would be more precise to acknowledge that, regardless of whether they co-occur within the same individual, both mistrust and credulity are recognized in the literature on epistemic trust as maladaptive epistemic stances stemming from traumatic experiences, particularly during childhood (as subsequently elaborated upon in the article). The current phrasing of the sentence might inadvertently convey the idea that mistrust serves as the precursor to credulity through the mechanism of epistemic hunger.

**(AR):** Thank you for addressing the ambiguity of the mentioned sentences. We have refined our argument in the paragraph to be more coherent and precise (line 66-74, page 4). In combination with rewording the subsequent paragraph, we hope this clarifies that both mistrust and credulity can result from adverse childhood experiences.

**(R#2):** Pag. 7, lines 149–168. To the point about hypotheses and aims, it would be helpful if the authors provided further clarification on their expectations concerning the relationship between sociodemographic characteristics and the three subscales of the ETMCQ.
**(AR):** Thank you for your comment. The results for the sociodemographic variables have been included in the results section to allow for classification and comparison with other studies and samples. As our main objective did not involve exploring these associations, we did not include any separate hypotheses regarding them. To clarify this, we paraphrased the related sentence in the Statistical Analysis section (line 253-255): “Exploratory, we analyzed associations between sociodemographic variables…”.

In the “Discussion” section of the manuscript:
**(R#2):** Page 22, lines 462-467. “The high correlative relationship between these two dimensions (mistrust and credulity) can be substantiated by the assumption that in the absence of ET, rapid shifts between credulity and hypervigilance could exist, therefore the two factors are closely related to each other. However, this interpretation raises the question of whether the mistrust subscale measures hypervigilance rather than persistent epistemic mistrust as a result of developmental adaptation to an adverse social environment”. The concept and term “epistemic mistrust” coincides with a rigid and all-encompassing epistemic hypervigilance, hence, the reason why the authors contrast mistrust with epistemic hypervigilance rather than epistemic vigilance is puzzling to me. Perhaps this section could benefit from further elaboration to clarify the rationale behind this comparison.
**(AR):** Thank you for your comment. We have now further elaborated this point to clarify the rationale. We referred to the Duschinsky and Foster’s (2021) discussion about the relationship between credulity and epistemic hypervigilance (e.g. see Figure 7.3 on p. 186). We now rephrased this part and added the following (in line 454-458): “The ETMCQ may be interpreted as capturing a two-dimensional construct. The subscales of mistrust (epistemic hypervigilance) and credulity represent two manifestations of the dysfunctional stance of epistemic mistrust. On the other hand, the functional stance of ET represents an adaptive balance between ET and epistemic vigilance.”

**(R#2):** Overall, I would like to highlight the need for a proofreading or a thorough revision of the English language, as to improve clarity and readability.
**(AR):** We have carefully revised the manuscript (especially the introduction and discussion), improving its precision, cohesiveness, and the readability.
