# Supplementary material for: Epistemic trust and associations with psychopathology: Validation of the German version of the Epistemic Trust, Mistrust and Credulity-Questionnaire (ETMCQ)
Source: PLoS One. 2024 Nov 14;19(11):e0312995. doi: 10.1371/journal.pone.0312995 (PMC11563411; doi:10.1371/journal.pone.0312995)
Supplement: Attachment — Submitted filename: Response to reviewers.docx [file pone.0312995.s009.docx]

Sabine Windmann
Academic Editor
PLOS ONE

Dear Ms. Windmann,

Herewith I am submitting a revised version of our manuscript **“Epistemic trust and associations with psychopathology: Validation of the German version of the Epistemic Trust, Mistrust and Credulity-Questionnaire (ETMCQ)”** to *PLOS ONE* on behalf of all authors.

We would like to thank you for giving us the opportunity to submit a revised version of our manuscript and also for your and the reviewer’s very helpful comments to improve the manuscript.

Please find below a point-by-point response to the reviewer’s comments, which we have incorporated in the manuscript (changes are highlighted in the main text).

The manuscript was prepared in accordance with PLOS ONE Style guidelines. The manuscript has not previously been published or is being considered for publication elsewhere. There was no conflict of interest for any of the authors of the article.

We hope that all revision points have been addressed satisfactorily and the manuscript now meets the high standards for publication in PLOS ONE and look forward to hearing from you.

Sincerely,

Anna-Maria Weiland

**Reply to the reviewer comments:**

**Editor (E):** Can you please review the statistical comment made by Reviewer 3 – an expert on SEM – about the skewed distributions of variables in the SEM? You may want to double-check with a statistician. Please modify as needed and/or provide evidence in the text or a supplement why skewness in the observed range is no problem for MLR. (Otherwise, page 13 is fine.)

**Author reply (AR):** Thank you for your comment. We reviewed the comment made by Reviewer 3 about the skewness in the SEM. You can find our answer in the response below.

**(E):** All reviewers comment on the surprisingly high correlation of credulity and mistrust. It is particularly surprising because these two concepts have been introduced as “opposites”! In view of your findings, then, how can you speak of a “validation”? Especially since the Campbell data show a different structure. I like your interpretation about the “rapid shifts”, but you need to explain why this should be the case only in your data. Could the elimination of the three low-loading items have led to that difference? What do the residual correlations say? (Again, consider a supplement). Can you check whether 2 or 3 factors are more suitable in your case? In fact, you speculate about this in the Discussion, so why not test the idea? Or test for invariance between your version and the one of Campbell? In whichever way you resolve the issue, please make sure this is being pointed out clearly in the Abstract. We want to ensure that readers of the Abstract understand that the underlying model of your scale is different from the original version.

**(AR):** Thank you for your important questions. To emphasize the differences between our results and Campbell's ETMCQ version more clearly, we have reformulated the conclusion in both the abstract and the discussion (line 456). In line 38-40 we stated out now: “The study offers initial empirical support for a revised 12-item self-report measure of epistemic trust and…” instead of “The study provides empirical evidence for the validity …”.

We revised the Introduction and clarified the definitions of the three epistemic stances observed in this study. Mistrust and credulity can be seen as opposite strategies in dealing with socially transmitted information, but both are dysfunctional, and both were associated with psychopathological phenomena.
In Line 476-479 we mentioned the possibility, that the differences between our factor structure and Campbell’s version may be an effect of the item reduction. We now have revised this sentence to emphasize this potential impact in particular regarding the extremely high correlation between mistrust and credulity.

We also added now the results of two CFA-models with 2 factors in Table 2 (line 371-372, p. 17). As you can see, model 4 with 3-factors, 12 items and correlated residuals remains still the best fitting model to our data. Therefore, in line 465-471 we included the following sentences in our discussion: “On the other hand, it is reasonable to conclude that mistrust and credulity may not be separate factors. This suggests that the ETMCQ may be interpreted as capturing a two-dimensional construct. […] However, subsequent testing of the two-factor models (models 5 and 6) to verify this assumption revealed a poorer fit to the data than the three-factor model (model 4).”

A table of the standardized residual correlations of model 4 were added in the supplement. Regarding the high correlation between mistrust and credulity we only found that two credulity items (item 05 and item 15) had noticeable residual correlations (> 3) with one mistrust item (item 04) and besides that, no all-over pattern could be found between mistrust items and credulity items. This result is underlined by a closer look at the modifications indices, where correlated residuals between item 04 and item 05/item 15 scored > 10.

And to compare the goodness of fit of the different models we tested in the CFA, we added the results of Likelihood ratio tests between our version of the ETMCQ (model 4) and the one of Campbell et al. (model 2), as well as between model 4 and the 2-factor model 6 (see line 373-375). Again, model 4 proofed to have a significant better model fit than the other two models. The same is indicated by a closer look at the Akaike information and Bayesian information criterion, as lower values indicate a better fit (see Table 2, line 378).
In conclusion, the three-factor structure is the optimal model in terms of content and factors for operationalizing the concept of epistemic trust for our data and at the same time a replication of our results in other German samples is needed.

**(E):** In the Introduction, although you describe credulity and mistrust as “opposites”, you sometimes speak of “positive associations between mistrust, credulity, and [other variables]”. The phrase is difficult to reconcile with your claim that credulity and mistrust are antagonistic. Can you clarify? If these are opposites, yet both correlate with variable X, I would expect the text to say: “positive associations between variable X and mistrust on the one hand and credulity on the other”, or: “associations that are positive between variable X and mistrust, but negative between variable X and credulity”. Likewise, in line 91, speaking of opposites, I would expect you to say: “both mistrust and credulity are considered to be adaptive responses…” Same holds for Hypothesis 3c and d on page 7, and summary of findings in the discussion, e.g. line 501. In fact, your hypotheses truly give away that you presumed from the outset that these two cannot be opposites! BTW any time you’d like to double-check your English, I recommend using deepl, in case you haven’t tried it yet. I think your language is very good though.

**(AR):** We are very grateful for your constructive feedback. In accordance with your recommendation, we have clarified our description of the associations between the two dysfunctional epistemic stances (mistrust and credulity) with psychopathological markers (line 75-77, 81-82, 92, 110-111, 498, 516). As previously stated, mistrust and credulity can be regarded as opposing strategies, yet both are maladaptive and linked to psychopathological phenomena. This conceptualization aligns with recent empirical studies on epistemic trust and the ETMCQ (Campbell et al., 2021; Liotti et al., 2023; Greiner et al., 2024; Asgarizadeh & Ghanbari, 2024). We believe this provides a rationale for our decision not to alter the wording in our hypotheses. In the discussion (line 545-547), we highlight the necessity for further investigation into the observed correlations between the subscales of the ETMCQ and maladaptive personality traits. This is to ascertain whether the identified patterns can be replicated in other samples.

**(E):** As Reviewer 1 points out, the work of Csibra and Gergely can be left out on page 1 or described only very briefly or maybe moved further down in the Introduction to substantiate the choice of childhood trauma measures. Generally, could you please have a closer look at the structure of the text on page 1 (BTW an unusually long paragraph!). First, you define ET. Then you jump to “natural pedagogy”, but without ever stating explicitly what this has to do with ET. [I think I know what you mean to say, but you don’t say it, and even if you did, it would disrupt the flow as you are leaving the level of conceptual definition and start speaking about ontology and adaptive functionality]. Then, starting with “However”, you suddenly go back to the level of conceptual definition. You say something along the lines of trust is not always beneficial, without saying why: Probably because it can open the door to being manipulated and exploited. If you’d say something like this explicitly, the following “therefore” would make much more sense! I hope you see what I mean… I am pointing this out so diligently only because the opening statements of a paper are so important.

**(AR):** Thank you for your comment. We have thoroughly revised the introduction, focusing on the definitions of trust, mistrust and credulity. As recommended, we have moved the section on Csibra and Gergely's natural pedagogy further back in the introduction and shortened it (line 66-71).

**(E):** Reviewers 1 and 3 were skeptical about study 2 for the low sample sizes. To address this point, you could perhaps state from the outset – perhaps on the basis of a power analysis – that your study design would allow you to detect only large effects. However, unlike Reviewer 3, I would not throw the study out, in the name of Open Science. After all, it is data; and you have made the attempt. Also, for that exact reason, I’d advise against calling it a “random result”. That phrasing seems to suggest that there was a positive finding that is based on chance. In reality, however, there no positive result and almost only chance. That’s why your only conclusion can be that you cannot interpret the pattern. Bayes analyses might help to see based on your data, how much more likely a null effect is compared to your hypothesized positive difference, but performing this analysis is not mandatory. However, please clearly state in the Abstract that you were “unsuccessful in finding” or “failed to find” (or something along those lines) the expected difference between the two groups. On a minor note, could you add one sentence in the Methods section about recruitment of the non-clinical sample?

**(AR):** Thank you for your valuable comment. Study 2 is the first tentative data from investigating the discriminative power of the ETMCQ and we are well aware of the small sample size and the need for replication. We agree with your feedback and decide to not throw out study 2. Instead, we followed your recommendations and clarified the phrasing about the findings regarding study 2 in the abstract and the discussion (line 35-37, 486-488, 492-494) to make clear, that “our study yielded inconclusive results regarding the differential strength of the ETMCQ” and emphasize “future research should endeavor to replicate this investigation with a larger sample size”. As we do not see any increase in information or change in the interpretation of the results, we have decided against the subsequent insertion of a post-hoc power analysis in the manuscript. Nevertheless, a post-hoc power analysis revealed a small power of β = 0.1 and a small effect size of *d* = 0.2.
We also clarified the sentence about recruitment of the clinical and the non-clinical sample (line 176-177).

**(E):** Could you add sample items to the description of the subscales of CAMSQ and LPFS?
**(AR):** We add sample items to the description of subscales of CAMSQ (Line 208-210) and LPFS (Line 229-231).

**(E):** Line 253: Should be adverbial: “Exploratorily”
**(AR):** Line 255: “Exploratory” is changed in “Exploratorily”.

**(E):** Line 174: Should be “diagnosis”
**(AR):** We corrected “diagnosis” in line 167.

**(E):** Table 2: Could you segregate model number from numbers of items further? Perhaps with a line break? à “Model 1: [line break] 15 items”
**(AR):** Thank you for your comment. We followed your recommendations and added a line break in Table 2.

**Reviewer #1: ID: PONE-D-23-22407R1**

**Reviewer #1 (R#1):** I have read the revised full text, and I have to say that this article is a very long story even though I understand the authors’ intention and core information, I still suggest, as I did in the first round, that the authors should streamline the language to lead the reader directly to the research topic and question and to cut out redundant information. The details of my question follow:
**(R#1):** 1. While the introduction effectively introduces the concept of ET and its relevance, some parts could be condensed for clarity and conciseness. For example, the discussion of Csibra and Gergely’s work on natural pedagogy could be summarized more succinctly.

**(AR):** Thank you again for your valuable comments. We followed your recommendations and further streamlined especially the first paragraph in the introduction. The discussion of Csibras and Gergely’s “natural pedagogy” is now summarized more succinctly and is removed to a later part in the introduction (Line: 66-71).

**(R#1):** 2. If “epistemic mistrust” and “credulity” are distinct constructs or if they are part of a broader ET construct? Please clarify this. Please provide more detailed information about empirical studies supporting the discussed concepts, especially regarding the associations between ET, mistrust, credulity, and psychopathology. This would strengthen the credibility of the arguments presented.

**(AR):** Thank you for your comment. Epistemic trust is a functional balance between trust and vigilance towards socially mediated information. Disturbances in this balance can result in either mistrust or credulity. These are phenomenologically distinct attitudes or strategies in dealing with socially mediated knowledge. However, both are dysfunctional and, as evidenced by empirical data, are associated with psychopathological markers. We have now revised the introduction, streamlined it and clarified the definitions of the three epistemic stances observed in this study. Furthermore, we now included two newly published ETMCQ-validation paper (Greiner et al., 2024; Asgarizadeh & Ghanbari, 2024) as well as other recently published empirical studies (Benzi et al., 2023; Parolin et al., 2023) to underscore our arguments regarding the associations between ET, mistrust, credulity, and difficulties in mentalizing (line 75-77), adverse childhood experiences (line 80-83), and psychopathology (line 89-96).

**(R#1):** 3. Some citations are presented as footnotes ([1], [2]), while others are within the text ([5, 6]). Please choose one format and apply it consistently.

**(AR):** Thank you for this comment. We do not know which footnotes you are referring to, as we do not use footnotes in the manuscript. In the text, we cite the reference number in square brackets as recommended by the submission guidelines of PLOS ONE.

**(R#1):** 4. I remain of the opinion that the size and composition of Sample 2 may have affected the reliability and generalization of the results and were not sufficient to support what this study was trying to explore.

**(AR):** We agree with your opinion about the sample size and its composition, which were indeed the two pivotal issues that we therefore address critically in the discussion. It should be noted, however, that interpretation of the results of Study 2 needs to be very cautious. However, as this is the first study to investigate the discriminative validity of the ETMCQ in a clinical and non-clinical population, and to follow the recommendations of the editor we keep these findings in the manuscript. As previously stated, we have rephrased some sentences in the abstract and discussion to clarify that our study yielded inconclusive results regarding the differential strength of the ETMCQ and to underscore the importance of future research replicating this investigation with a larger sample size (line 35-37, 486-488, 492-494).

**(R#1):** 5. While you mention data cleaning, consider providing a brief overview of the specific steps taken to clean and prepare the data for analysis, as this can impact the validity and reliability of the results. Please consider including any robustness checks or sensitivity analyses conducted to ensure the robustness of the study findings.

**(AR):** Our study is part of a larger research project, and a revised version of the associated article (cited in line 158) has been submitted. In the “Statistical Analysis” section, there is a reference to the preregistration (<https://doi.org/10.23668/psycharchives.5406>) and a reference to the associated article to make the data cleansing strategies available (line 250). Beyond that we now added a brief description of the results of the specific steps to clean the data: “In the self-report sample 622 participants completed the study and passed the attention check item. After that, 19 participants from sample 1 were excluded based on the relative speed index (Leiner, 2019). Finally, the long string method was used to detect careless respondents (Curran, 2015), which resulted in the exclusion of 19 additional participants in sample 1 (*N* = 584).”(line 250-253).

**(R#1):** 6. I’m not very satisfied with the existing table presentation; the words are squished together, which detracts from the basic clarity.

**(AR):** Thank you for pointing this out. We revised table 2 and added a line break and clarified the description of the different CFA-models.

**(R#1):** 7. In the discussion section, the authors mention the consistency and discrepancies between the findings and prior theories, but the reasons for these discrepancies could be explored in more depth. For example, the authors could have discussed the focus of different theoretical frameworks for explaining trust, mistrust, and gullibility behaviors, and whether these focuses explain the findings differently.

**(AR):** Thank you for your comments. While the discussion of different theoretical frameworks is undoubtedly valuable, it is beyond the scope of this particular work, as the aim was to test the operationalization of one construct. A brief reference to this topic was made in the discussion, as indicated in lines 628 ff.: “It is important to address the existing criticism of the concept of ET, including the imprecise use of the terms trust and mistrust, as well as the lack of classification of ET in the context of interdisciplinary trust research [67]. Therefore, future research should aim to clarify the differences and connections between trust concepts, including its relationship to interpersonal trust, as suggested by Botsford and Renneberg [68].” Furthermore, we briefly discussed our findings and the discrepancies with Campbell et al. with the focus on different theoretical considerations regarding epistemic trust (ET as a transdiagnostic resilience factor vs. ET as a neutral human default mode; see line 558-561).

**(R#1):** Overall, I consider this study to be open to scrutiny in terms of quality and needs significant revision before it can be published. I am sorry to reject the manuscript and look forward to future opportunities to see more of your excellent work.

**(AR):** We are grateful and understand your critical feedback. We revised the manuscript and hope your critical points are sufficiently addressed.

**Reviewer #3: ID: PONE-D-23-22407R1**

**Reviewer #3 (R#3):** SEM analysis: p13. The authors speak of a 5-factor model they have fit, and then they seem to talk about sth else and report a very high value of multiv. skewness for a CFA of the CAMSQ scale. The value of the multiv. skewness appears way too high to simply continue with an analysis of a SEM model. MLR estimation probably cannot cope with having such a large value of skewness in the data. This makes the results very doubtful in its entirety. The authors should clearly report on the skewness of the separate indicator variables. Then, depending on whether that there continue to be some extremely skewed indicators, they cannot simply proceed with the SEM analysis. MLR estimation still requires skewness to lie within some moderate range. So, it has to be clarified if one could at all proceed with SEM from here on. A problem is also the poor statistical model fit of model 1. What explains it? Is the theory wrong, or does the sample have characteristics that would lead to it? What does the residual correlation matrix say? For a revision of the paper, these questions

should be clearly addressed.

**(AR):** Regarding the multivariate skewness, there might have been a misunderstanding, and we want to clarify that the preconditions to conduct an SEM with the robust estimation method MLR were fulfilled. We reported the results of the Mardia’s Test and therefore reported the test statistic and p-value for multivariate skewness and kurtosis. As you recommended and for clarity reasons, we now added the following sentence (line 302-304): “The univariate skew values for each indicator variable were between -1 and +1 (self-certainty: - 0.59; other-certainty: -0.23; trust: -0.67; mistrust: -0.16; credulity: 0.46).”.

In regard to the inadequate model fit of Model 1, it appears to be attributable to either a theoretical or an item-based explanation. In all three available validation studies conducted in languages other than English, the original 15-item model proposed by Campbell et al. demonstrated an inadequate model fit (Liotti et al., 2023; Greiner et al., 2024; Asgarizadeh & Ghanbari, 2024). All three of the aforementioned validation studies excluded one or three items (Item 11 by Liotti et al., item 14 by Asgarizadeh & Ghanbari, item 1, 3 and 6 by Greiner et al.). Our study yielded results that were partially overlapping but also somewhat distinct, as we excluded items 3, 6, and 14. In the discussion, we now emphasize the “potential influence of cultural and linguistic factors on the discrepancies observed in the separate validation studies” (line 479-482). In addition to emphasizing the importance of considering cultural influences in general (see lines 624-626).
The residual correlation matrix of model 1 underscores the inadequate model fit to the data and reveals high residual correlations especially for the excluded items 6 and 14 (see S5 Table A in the newly added supplement).

**(R#3):** Model 2: How large were the correlations among the residuals? The interpretation of the results depend on this.

**(AR):** As illustrated in the table of standardized residual correlations for Model 2, there are notable instances of high correlation among residuals, in addition to the residual correlations incorporated from the original model by Campbell et al.. The most critical instances are observed within a range of 3 to 7. Align with the chosen method for improving model fit by Campbell et al., we decided to remove low loading items.

**(R#3):** Model 4: Factor 2 and 3 were highly correlated, which speaks perhaps for 2-factor model rather than 3 factors. Could that be a consequence of the shortening of the questionnaire? The shortening appears to have been made based on the original data (it’s just data-fitting by changing the instrument) without cross-validation. An actual argument for a replication of Campbell’s factor structure would have to go into a cross-validation here, by pulling another sample and fitting it both to the 15-item version and the shortened 12-item version.

**(AR):** Thank you for your question and comment. We agree that the high correlation between mistrust and credulity is striking. We have taken up the consideration of whether this is due to the shortening of the mistrust scale in the discussion (line 476-479). We have now additionally tested the idea of a 2-factor model and added the results (see table 2 line 378). As you can see our model 4 with 3 Factors still remains the best fitting model to our data. In the Discussion in line 479-482 we emphasize that the factor structure for the German version needs to be further investigated and that our findings also need to be further tested.

**(R#3):** Discriminant validity: The sample size for sample 2 is just too small right from the start to address the research question. I believe it should be taken out from the manuscript.

**(AR):**  As described above, we agree with your opinion about the sample size and its composition, which were indeed the two pivotal issues that we therefore address critically in the discussion. It should be noted, however, that interpretation of the results of Study 2 needs to be very cautious. However, as this is the first study to investigate the discriminative validity of the ETMCQ in a clinical and non-clinical population, and to follow the recommendations of the editor we keep these findings in the manuscript. As previously stated, we have rephrased some sentences in the abstract and discussion to clarify that our study yielded inconclusive results regarding the differential strength of the ETMCQ and to underscore the importance of future research replicating this investigation with a larger sample size (lines 35-37, 486-488, 492-494).
